# Association of sleep quality with temperament among one-month-old infants in The Japan Environment and Children’s Study

**DOI:** 10.1371/journal.pone.0274610

**Published:** 2022-09-14

**Authors:** Kimiyo Kikuchi, Takehiro Michikawa, Seiichi Morokuma, Norio Hamada, Yoshiko Suetsugu, Kazushige Nakahara, Kiyoko Kato, Masafumi Sanefuji, Eiji Shibata, Mayumi Tsuji, Masayuki Shimono, Toshihiro Kawamoto, Shouichi Ohga, Koichi Kusuhara, The Japan Environment and Children’s Study Group

**Affiliations:** 1 Department of Health Sciences, Graduate School of Medical Sciences, Kyushu University, Fukuoka, Japan; 2 Department of Environmental and Occupational Health, School of Medicine, Toho University, Tokyo, Japan; 3 Research Center for Environment and Developmental Medical Sciences, Kyushu University, Fukuoka, Japan; 4 Department of Obstetrics and Gynecology, Graduate School of Medical Sciences, Kyushu University, Fukuoka, Japan; 5 Department of Pediatrics, Graduate School of Medical Sciences, Kyushu University, Fukuoka, Japan; 6 Regional Center for Japan Environment and Children’s Study, University of Occupational and Environmental Health, Kitakyushu, Japan; 7 Department of Obstetrics and Gynecology, School of Medicine, University of Occupational and Environmental Health, Kitakyushu, Fukuoka, Japan; 8 Department of Environmental Health, School of Medicine, University of Occupational and Environmental Health, Kitakyushu, Fukuoka, Japan; 9 Department of Pediatrics, School of Medicine, University of Occupational and Environmental Health, Kitakyushu, Fukuoka, Japan; Kyung Hee University School of Medicine, REPUBLIC OF KOREA

## Abstract

This study aimed to examine the association between infant sleep quality and temperament in one-month-old infants using a large cohort study data. We used data from the Japan Environment and Children’s Study, a cohort study which follows around 100,000 women from pregnancy until their children’s development. The mothers were asked about their infants’ sleep and temperament using a structured questionnaire. Frequent crying (adjusted odds ratio [AOR]: 1.05, 95% confidence interval [CI]: 1.00–1.10) and intense crying (AOR: 1.19, 95% CI: 1.13–1.25) were positively associated with longer sleep periods during the day than at night. Female infants with longer daytime sleep periods than that at nighttime were more likely to cry frequently (AOR: 1.11, 95% CI: 1.04–1.20). Parous women with infants who had frequent night awakening believed their infants cried more intensely (AOR: 1.17, 95% CI: 1.03–1.31). The study demonstrated a specific association between sleep quality and temperament in one-month-old infants. Based on the results of this study, further sleep intervention studies are required to improve infant temperament.

## Introduction

The importance of temperament in infants has been attracting attention. Temperament is complexly affected by genetic and environmental factors [1]. Infant temperament is linked to underlying neural networks [2] and refers to the initial state of psychopathological and behavioral development [3]. A correlation between early development through temperament and attention-deficit/hyperactivity disorder at a later age has been particularly discussed [4,5].

Infant temperament may be influenced by sleep quality. Healthy sleep is defined by its appropriateness in duration, timing, quality, and absence of disturbances [6,7]. Previous studies have demonstrated various sleep qualities related to temperament during the first year of life, such as sleep duration and night awakening [8–10]. In particular, shorter nighttime sleep periods and frequent night awakenings were associated with negative or difficult infant temperament [10,11]. This may be explained by the sleep difficulties that inhibit neuropsychological functioning [12,13]. However, another study has reported weak to no association between sleep and temperament [11]. Thus, the exact nature of this relation remains ambiguous [14].

To the best of our knowledge, no study has been conducted yet to thoroughly investigate the relationship between infant sleep quality and temperament, especially in one-month-old infants. However, it is essential to elucidate this relationship in early infancy. Once this relationship is clarified, early intervention in infant sleep may help control later temperament problems, thus preventing developmental problems. Hence, this study aims to examine the association between sleep quality and temperament in one-month-old infants using data from a large cohort study.

## Methods

### Study design and participants

**Fig 1:**
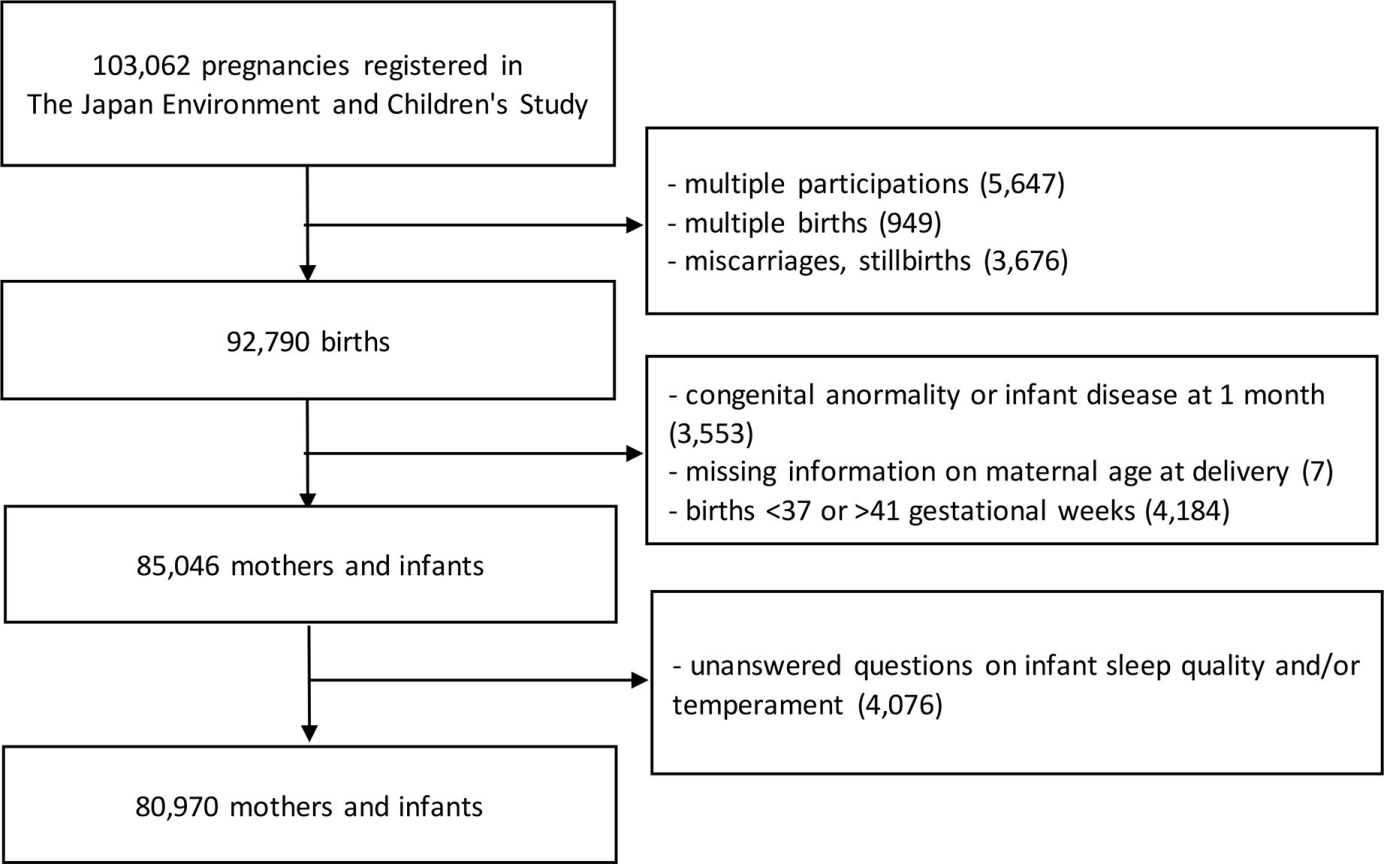
Flow diagram of the selection process of study participants.

This study is a cross-sectional study conducted as part of Japan’s nationwide prospective birth cohort study, the Japan Environment and Children’s Study (JECS), which aims to elucidate the environmental factors affecting children’s health and development. The study was registered in the UMIN Clinical Trials Registry (number UMIN000030786), while the protocol and baseline profile of the participants were reported elsewhere [15,16]. JECS follows up on around 100,000 pregnant women and their children until the children reach 13 years of age. Pregnant women were recruited between January 2011 and March 2014 from 15 Regional Centres throughout Japan. In this study, we analyzed maternal and infant data, excluding data from cases of multiple participating pregnant women, miscarriages or stillbirths, missing information on maternal age at delivery, multiple births, births at <37 or >41 gestational weeks, congenital abnormality or disease in one-month-old infants, and unanswered questions on infant sleep quality or temperament (Fig 1). In this study, the definition of a “one-month-old infant” was an infant 30 or 31 days after birth.

### Data collection

After registration, the pregnant women answered self-administered questionnaires in the first and second/third trimesters and the first month after birth (median: 30 (27–33) days). The medical records of the infants at birth were also transcribed and collected by physicians, midwives/nurses, and/or Research Co-ordinators.

#### Infant sleep

The infant’s sleep quality was identified by the awakening frequency and the length of sleep, which were indicated as predictors of children’s developmental problems [17]. Mothers ticked the infants’ sleep time on sleep/wake the day before the data collection date, marking the infant’s sleep every 30 minutes. The frequency of awakening was assessed by the number of nocturnal awakenings between 8:00 pm and 7:59 am before the day of data collection. We set ≥5 awakenings as the points that define higher frequency because the awakening frequency ranges from 1.0 to 5.0 between 8:00 pm and 7:59 am for 2-week-old neonates [18]. Comparison of sleep duration was assessed by the length of sleep periods during nighttime (8:00 pm to 7:59 am) and that during daytime (8:00 am to 7:59 pm). We defined longer daytime sleep periods than that during nighttime as unusual.

#### Infant temperament

Infant temperament was assessed by the mothers’ responses to the questions on their infants’ mood, crying degree, and crying intensity, which related to children’s developmental disorder [17]. Infant mood was identified by the question, "*Frequency of having difficulty while holding the baby due to his/her affection and/or behavior (e*.*g*., *crying*, *bending backwards*, *etc*.*)*.*"* Answer options included "*often*,” "*sometimes*,” "*seldom*," and "*never*." *Often* was categorized as a neonatal tendency to be in a "bad mood." The question to assess the degree of crying was "*Intensity and frequency of crying (baby)"* Answer options included "*quite often and long*,” "*sometimes and short*," and "*hardly*." *Quite often and long* was categorized as "frequent crying, for long periods." The question to assess crying intensity was "*I have trouble calming down my crying baby"* The question was answerable by yes/no. “*Yes”* was categorized as "intense crying." All these categorizations were defined in previous studies [19,20].

#### Covariates

In addition to infant sleep and temperament data, other covariates were included in the analyses following previous studies that assessed factors associated with infant sleep [20–25]. Maternal data included maternal age at delivery, parity or any previous childbirth experience, sleep duration of the mother during pregnancy, smoking habits during pregnancy, marital status, maternal educational background, household income, and postnatal depressive symptoms (Edinburgh Postnatal Depression Scale [26,27]: a score of ≥9 out of 30 was defined as having depressive symptoms in Japan [28]). Infant data included the gestational week at birth, birth weight (<10^th^ percentile of birth weight standards by gestational age is categorized as small for gestational age for Japanese neonates [29]), infant sex, and feeding status.

### Statistical analyses

First, we descriptively analyzed the data according to infant sleep quality categories and identified the cutoffs as ≥5 nighttime awakenings and longer daytime sleep periods than at nighttime. We then used the Chi-square test to examine the relationship between infant sleep quality and temperament [30]. Then, we conducted logistic regression analyses to assess the association between sleep quality and temperament [31]. We set two dependent variables, *≥5 nighttime awakenings* and *longer daytime sleep periods than at nighttime*, and three independent variables, *bad mood*, *frequent crying for a long period*, and *intense crying*. The association between each dependent variable and each independent variable was examined by adjusting for covariates. We then assessed the association between infant sleep quality and temperament as stratified by sex or mother’s parity. We performed analyses in the models with only maternal age and infant and maternal covariates. We also assessed the association of infant’s sleep quality and temperament stratified by infant’s sex or mother’s parity, which have been identified as factors associated with infant temperament [19,32]. Statistical evidence of the effect modification was checked using cross-product terms of exposures and infant’s sex (or parity). All data analyses were performed using STATA version 16.1 (StataCorp LLC. College Station, TX, USA). The dataset used for this study was the jecs-an-20180131 dataset released in March 2018.

### Ethical approval

The JECS protocol was reviewed and approved by the Ministry of the Environment’s Institutional Review Board on Epidemiological Studies and the Ethics Committees of all participating institutions. The study has been conducted under the Declaration of Helsinki and Japan’s Ethical Guidelines for Epidemiological Research issued by the Ministry of Education, Culture, Sports, Science, and Technology and the Ministry of Health, Labor, and Welfare, Japan. Written informed consent was obtained from all participants.

## Results

Of 103,062 pregnancies registered in the Japan Environment and Children’s Study (JECS), we analyzed 80,970 eligible maternal and infant dyad data for this study. As shown in Table 1, there were 5,158 infants (6.4%) categorized to have *≥5 nighttime awakenings* and 15,616 infants (19.3%) categorized to have *longer daytime sleep periods than at nighttime*. Both had a median sleep duration of 15.5 hours (Interquartile range: 14.5–17.0). Regarding the temperaments, bad mood, frequent crying for a long duration, and intense crying was observed in 6.2%, 17.2%, and 19.5% of infants, respectively.

**Table 1:** Characteristics of the study participants.

	Total population (n = 80,970)		Five or more awakenings during the night				Sleeping longer during the day than at night			
			No (n = 75,812)		Yes (n = 5,158)		No (n = 65,354)		Yes (n = 15,616)	
	n^a^	(%)	n^a^	(%)	n^a^	(%)	n^a^	(%)	n^a^	(%)
**Maternal characteristics**										
**Age at delivery (years)**										
< 25	354	6.9	7,472	9.9	354	6.9	6,073	9.3	1,753	11.2
25–29	1,357	26.3	21,150	27.9	1,357	26.3	18,215	27.9	4,292	27.5
30–34	1,943	37.7	26,854	35.4	1,943	37.7	23,400	35.8	5,397	34.6
≥ 35	1,504	29.2	20,336	26.8	1,504	29.2	17,666	27.0	4,174	26.7
Parity										
0	1,839	35.8	33,381	44.2	1,839	35.8	27,112	41.6	8,108	52.1
≥ 1	3,293	64.2	42,146	55.8	3,293	64.2	37,991	58.4	7,448	47.9
**Smoking habits**										
Never smoked	3,018	58.8	44,256	58.6	3,018	58.8	38,346	58.9	8,928	57.4
Ex-smokers who quit before pregnancy	1,294	25.2	17,652	23.4	1,294	25.2	15,323	23.5	3,623	23.3
Smokers during early pregnancy	824	16.0	13,634	18.1	824	16.0	11,447	17.6	3,011	19.4
**Marital status**										
Married	4,919	96.4	71,653	95.6	4,919	96.4	61,930	95.8	14,642	94.8
Unmarried	156	3.1	2,719	3.6	156	3.1	2,195	3.4	680	4.4
Divorced/widowed	28	0.6	614	0.8	28	0.6	518	0.8	124	0.8
**Educational background (years)**										
< 10	210	4.1	3,388	4.5	210	4.1	2,824	4.4	774	5.0
10–12	1,470	28.8	23,369	31.2	1,470	28.8	19,924	30.9	4,915	31.8
13–16	3,349	65.6	46,997	62.8	3,349	65.6	40,791	63.2	9,555	61.9
≥ 17	78	1.5	1,129	1.5	78	1.5	1,012	1.6	195	1.3
**Household income (million Japanese-yen/year)**										
< 2	267	5.5	3,854	5.5	267	5.5	3,251	5.4	870	6.0
2 to < 4	1,549	32.1	24,132	34.4	1,549	32.1	20,595	34.0	5,086	35.3
4 to < 6	1,673	34.7	23,180	33.1	1,673	34.7	20,074	33.2	4,779	33.2
6 to < 8	756	15.7	11,322	16.1	756	15.7	9,915	16.4	2,163	15.0
8 to < 10	334	6.9	4,705	6.7	334	6.9	4,156	6.9	883	6.1
≥ 10	243	5.0	2,945	4.2	243	5.0	2,568	4.2	620	4.3
**Postpartum depressive symptoms at 1 month after delivery assessed by Edinburgh Postnatal Depression Scale**										
No (score < 8)	4,402	86.3	64,255	85.9	4,402	86.3	55,794	86.5	12,863	83.6
Depressive (score ≥ 9)	698	13.7	10,554	14.1	698	13.7	8,733	13.5	2,519	16.4
**Sleep duration during pregnancy (hours)**										
< 6	245	4.8	3,669	4.9	245	4.8	2,999	4.7	915	5.9
6 to <7	765	15.0	11,194	15.0	765	15.0	9,297	14.4	2,662	17.3
7 to <8	1,523	29.9	23,345	31.2	1,523	29.9	20,025	31.1	4,843	31.4
8 to <9	1,441	28.3	21,295	28.5	1,441	28.3	18,591	28.8	4,145	26.9
9 to <10	801	15.7	10,487	14.0	801	15.7	9,389	14.6	1,899	12.3
> = 10	324	6.4	4,842	6.5	324	6.4	4,202	6.5	964	6.3
**Infant characteristic**										
**Gestational week**										
37	575	11.2	7,213	9.5	575	11.2	6,006	9.2	1,782	11.4
38	1,385	26.9	17,266	22.8	1,385	26.9	14,748	22.6	3,903	25.0
39	1,564	30.3	22,372	29.5	1,564	30.3	19,462	29.8	4,474	28.7
40	1,264	24.5	21,466	28.3	1,264	24.5	18,623	28.5	4,107	26.3
41	370	7.2	7,495	9.9	370	7.2	6,515	10.0	1,350	8.6
**Small for gestational**										
No	4,751	92.6	70,062	92.8	4,751	92.6	60,465	92.9	14,348	92.2
Yes	381	7.4	5,465	7.2	381	7.4	4,638	7.1	1,208	7.8
**Infant sex**										
Male	2,818	54.6	38,393	50.6	2,818	54.6	33,466	51.2	7,745	49.6
Female	2,340	45.4	37,419	49.4	2,340	45.4	31,888	48.8	7,871	50.4
**Feeding status**										
Breastfeeding	3,366	66.4	39,134	52.8	3,366	66.4	34,893	54.6	7,607	49.9
Partial breastfeeding	1,627	32.1	32,103	43.3	1,627	32.1	26,699	41.8	7,031	46.1
Formula feeding	74	1.5	2,898	3.9	74	1.5	2,356	3.7	616	4.0
Sleep duration (hours), median (IQR)	15.5 (14–17)		15.5 (14–17)		15.5 (14.5–17)		15.5 (14–17)		15.5 (14–17)	
Sleep duration (hours) during the night (20:00 to 7:59), median (IQR)	8.5 (7.5–9.5)		8.5 (7.5–9.5)		8.5 (7.5–9)		8.5 (8–9.5)		7 (6–8)	
Sleep duration (hours) during the day (8:00 to 19:59), median (IQR)	7 (6–8)		7 (6–8)		7.5 (6.5–8.5)		7 (5.5–7.5)		8.5 (7.5–9)	
Number of awakenings during the night, median (IQR)	3 (2–3)		n.a.		n.a.		3 (2–3)		3 (2–3)	

^a^Subgroup totals do not equal the overall number because of missing data.

IQR:Interquartile range; n.a: Not applicable.

### Relationship between infant sleep quality and temperament using the Chi-square test

Table 2 demonstrates the relationship between infant sleep quality and temperament using the Chi-square test. Significant relationships were observed between frequent nighttime awakening and bad mood (p < 0.001) or intense crying (p = 0.001). There was a relationship between longer daytime sleep periods than at nighttime and bad mood, frequent crying, or intense crying (all, p < 0.001).

**Table 2:** Relationship between infant sleep quality and temperament in one-month-olds according to the Chi-square test.

	Total of respondents to the temperament questions		Infants who had five or more awakenings during the night				*Chi-square test P*-value	Infants who slept longer during the day than at night				*Chi-square test P*-value
			No		Yes			No		Yes		
	n	%	n	%	n	%		n	%	n	%	
**Bad mood**												
**No**	75,817	93.8	70,941	93.7	4,876	94.7	< 0.001	61,378	94.1	14,439	92.6	< 0.001
**Yes**	5,022	6.2	4,747	6.3	275	5.3		3,866	5.9	1,156	7.4	
**Frequent crying**												
**No**	66,792	82.8	62,495	82.7	4,297	83.6	0.114	54,214	83.2	12,578	80.8	< 0.001
**Yes**	13,913	17.2	13,068	17.3	845	16.4		10,929	16.8	2,984	19.2	
**Intense crying**												
**No**	64,982	80.5	60,745	80.4	4,237	82.3	0.001	53,114	81.5	11,868	76.3	< 0.001
**Yes**	15,748	19.5	14,839	19.6	909	17.7		12,053	18.5	3,695	23.7	

### Association of infant sleep quality with temperament by multivariate analyses

As shown in Table 3, a bad mood in infants (adjusted odds ratio [AOR]: 0.84, 95% confidence interval [CI]: 0.74–0.96) and intense crying (AOR: 0.88, 95% CI: 0.82–0.95) were negatively associated with frequent nighttime awakening when adjusted for maternal age. However, they were not associated with it after adjusting for other covariates. We also identified the adjusted odds ratio of having longer daytime sleep periods than at nighttime, with a bad mood in infants (AOR: 1.07, 95% CI: 0.99–1.16), frequent crying (AOR: 1.05, 95% CI: 1.00–1.10), and intense crying (AOR: 1.19, 95% CI: 1.13–1.25).

**Table 3:** Logistic regression analysis of association of infant sleep quality with temperament in one-month-olds.

	Total of respondents to the sleep quality questions	No. of respondents to the temperament questions		Crude model			Maternal age adjusted model			Infant and maternal attributes adjusted model^†^		
		n	%	OR	95% CI		AOR	95% CI		AOR	95% CI	
**Exposure: awakening for five or more times during the night**												
**Bad mood**												
No	75,688	4,747	6.3	Reference			Reference			Reference		
Yes	5,151	275	5.3	0.84	0.74	0.96	0.84	0.74	0.96	0.97	0.85	1.11
**Frequent crying**												
No	75,563	13,068	17.3	Reference			Reference			Reference		
Yes	5,142	845	16.4	0.94	0.87	1.01	0.94	0.87	1.01	1.07	0.98	1.16
**Intense crying**												
No	75,584	14,839	19.6	Reference			Reference			Reference		
Yes	5,146	909	17.7	0.88	0.82	0.95	0.88	0.82	0.95	1.04	0.96	1.13
**Exposure: sleeping longer during the day than at night**												
**Bad mood**												
No	65,244	3,866	5.9	Reference			Reference			Reference		
Yes	15,595	1,156	7.4	1.27	1.19	1.36	1.27	1.19	1.36	1.07	0.99	1.16
**Frequent crying**												
No	65,143	10,929	16.8	Reference			Reference			Reference		
Yes	15,562	2,984	19.2	1.18	1.13	1.23	1.18	1.13	1.23	1.05	1.00	1.10
**Intense crying**												
No	65,167	12,053	18.5	Reference			Reference			Reference		
Yes	15,563	3,695	23.7	1.37	1.32	1.43	1.37	1.31	1.43	1.19	1.13	1.25

CI: Confidence interval; AOR: Adjusted odds ratio.

^†^Adjusted for maternal age at delivery, parity, sleep duration during pregnancy, smoking habits, marital status, maternal educational background, household income, postpartum depressive symptoms, gestational age at birth,small for gestational age, infant sex, and feeding status.

### Sex-stratified association of infant sleep quality with temperament by multivariate analyses

Table 4 shows the sex-stratified association of sleep quality in infants with temperament. The analysis demonstrates effect modification by sex in the association of longer daytime sleep periods with frequent crying (p = 0.02). In conclusion, female infants with longer daytime sleep periods were more likely to cry frequently (AOR: 1.11, 95% CI: 1.04–1.20). Longer daytime sleep periods than at nighttime were associated with intense crying in both female (AOR: 1.24, 95% CI: 1.16–1.33) and male infants (AOR: 1.14, 95% CI: 1.07–1.22) and no effect modification by sex was observed (p = 0.08).

**Table 4:** Infant sex-stratified analysis of the association between infant sleep quality and temperament in one-month-olds.

	Male infants									Female infants									
Respondents to the sleep quality questions		Respondents to the temperament questions		Maternal age adjusted model			Infant and maternal attributes adjusted model^†^			Respondents to the sleep quality questions	Respondents to the temperament questions		Maternal age adjusted model			Infant and maternal attributes adjusted model^†^			P-value for effect modification by sex
		n	%	AOR	95% CI		AOR	95% CI			n	%	AOR	95% CI		AOR	95% CI		
**Exposure: awakening for five or more times during the night**																			
**Bad mood**																			
No	38,327	2,655	6.9	Reference			Reference			37,361	2,092	5.6	Reference			Reference			
Yes	2,814	153	5.4	0.74	0.65	0.92	0.91	0.76	1.09	2,337	122	5.2	0.93	0.77	1.12	1.06	0.86	1.31	0.30
**Frequent crying**																			
No	38,272	7,122	18.6	Reference			Reference			37,291	5,946	15.9	Reference			Reference			
Yes	2,810	495	17.6	0.93	0.84	1.03	1.08	0.97	1.21	2,332	350	15.0	0.93	0.83	1.04	1.04	0.92	1.18	0.58
**Intense crying**																			
No	38,275	7,618	19.9	Reference			Reference			37,309	7,221	19.4	Reference			Reference			
Yes	2,812	487	17.3	0.85	0.76	0.94	1.02	0.92	1.15	2,334	422	18.1	0.92	0.83	1.03	1.06	0.94	1.19	0.72
**Exposure: sleeping longer during the day than at night**																			
**Bad mood**																			
No	33,408	2,183	6.5	Reference			Reference			31,836	1,683	5.3	Reference			Reference			
Yes	7,733	625	8.1	1.26	1.15	1.38	1.07	0.96	1.18	7,862	531	6.8	1.30	1.17	1.44	1.07	0.96	1.20	0.99
**Frequent crying**																			
No	33,364	6,073	18.2	Reference			Reference			31,779	4,856	15.3	Reference			Reference			
Yes	7,718	1,544	20.0	1.12	1.06	1.20	1.00	0.93	1.07	7,844	1,440	18.4	1.25	1.17	1.33	1.11	1.04	1.20	0.02
**Intense crying**																			
No	33,374	6,304	18.9	Reference			Reference			31,793	5,749	18.1	Reference			Reference			
Yes	7,713	1,801	23.4	1.31	1.23	1.39	1.14	1.07	1.22	7,850	1,894	24.1	1.44	1.36	1.53	1.24	1.16	1.33	0.08

CI: Confidence interval; AOR: Adjusted odds ratio.

*p<0.05.

^†^Adjusted for maternal age at delivery, parity, sleep duration during pregnancy, smoking habits, marital status, maternal educational background, household income, postpartum depressive symptoms, gestational age at birth, small for gestational age, infant sex, and feeding status.

### Parity-stratified association between infant sleep quality and temperament by multivariate analyses

Table 5 shows the parity-stratified association of infant sleep quality with temperament. Effect modification by parity was detected in the association of frequent awakening with intense crying (p = 0.02). Parous women who had infants with frequent awakening felt that their infants cried more intensely (AOR: 1.17, 95% CI: 1.03–1.31). Although effect modification of parity was not identified, frequent night awakening in infants was associated with frequent crying by parous women (AOR: 1.13, 95% CI: 1.01–1.28). Longer daytime sleep periods than at nighttime were associated with bad mood by parous women (AOR: 1.20, 95% CI: 1.02–1.42) and with frequent crying by nulliparous women (AOR: 1.08, 95% CI: 1.02–1.15). Longer daytime sleep periods than at nighttime were also associated with intense crying by both nulliparous (AOR: 1.20, 95% CI: 1.13–1.27) and parous women (AOR: 1.17, 95% CI: 1.07–1.27).

**Table 5:** Parity-stratified analysis of the association between infant sleep quality and temperament in one-month-olds.

	Nulliparae									Parous women									P-value for effect modification by parity
Respondents to the sleep quality questions		Respondents to the temperament questions		Maternal age adjusted model			Infant and maternal attributes adjusted model^†^			Respondents to the sleep quality questions	Respondents to the temperament questions		Maternal age adjusted model			Infant and maternal attributes adjusted model^†^			
		n	%	AOR	95% CI		AOR	95% CI			n	%	AOR	95% CI		AOR	95% CI		
**Exposure: awakening for five or more times during the night**																			
**Bad mood**																			
No	33,329	3,691	11.1	Reference			Reference			42,074	1,029	2.5	Reference			Reference			
Yes	1,837	203	11.1	0.98	0.85	1.14	1.03	0.87	1.20	3,288	71	2.1	0.88	0.69	1.12	0.83	0.64	1.09	0.14
**Frequent crying**																			
No	33,254	8,226	24.7	Reference			Reference			42,026	4,783	11.4	Reference			Reference			
Yes	1,833	433	23.6	0.93	0.83	1.04	0.99	0.88	1.12	3,283	409	12.5	1.10	0.99	1.23	1.13	1.01	1.28	0.13
**Intense crying**																			
No	33,287	10,435	31.4	Reference			Reference			42,012	4,340	10.3	Reference			Reference			
Yes	1,839	538	29.3	0.89	0.80	0.99	0.95	0.85	1.06	3,281	369	11.3	1.09	0.98	1.22	1.17	1.03	1.31	0.02
**Exposure: sleeping longer during the day than at night**																			
**Bad mood**																			
No	27,071	2,965	11.0	Reference			Reference			37,922	880	2.3	Reference			Reference			
Yes	8,095	929	11.5	1.06	0.98	1.14	1.04	0.95	1.13	7,440	220	3.0	1.28	1.10	1.48	1.20	1.02	1.42	0.08
**Frequent crying**																			
No	27,011	6,564	24.3	Reference			Reference			37,883	4,311	11.4	Reference			Reference			
Yes	8,076	2,095	25.9	1.10	1.03	1.16	1.08	1.02	1.15	7,426	881	11.9	1.04	0.96	1.13	1.00	0.92	1.09	0.21
**Intense crying**																			
No	27,044	8,189	30.3	Reference			Reference			37,872	3,815	10.1	Reference			Reference			
Yes	8,082	2,784	34.5	1.22	1.15	1.28	1.20	1.13	1.27	7,421	894	12.1	1.22	1.13	1.31	1.17	1.07	1.27	0.65

CI: Confidence interval; AOR: Adjusted odds ratio

*p<0.05.

^†^Adjusted for maternal age at delivery, parity, sleep duration during pregnancy, smoking habits, marital status, maternal educational background, household income, postpartum depressive symptoms, gestational age at birth, small for gestational age, infant sex, and feeding status.

## Discussion

To the best of our knowledge, this study is the first to demonstrate the association between sleep quality and temperament among one-month-old infants. Our previous study showed that sleep problems in pregnant women were related to the temperament of one-month-old infants. The results of this study may add further knowledge on the possible improvement of infant temperament through the betterment of infant sleep quality.

According to the present study results, a longer daytime sleep period in infants than at night was a factor associated with infant temperament, frequent crying, and intense crying. Although the study population was not the same, a previous study reported a similar result demonstrating that infants with difficult temperaments tend to have less nighttime sleep than daytime sleep [14]. A previous study suggested that intense crying in young infants could be determined by the developmental interaction of sleep homeostatic and circadian processes and suggested that excessive crying may indicate delayed neurological maturation [33]. These results are limited because the causal relationship cannot be confirmed in this study; however, they suggest the possibility that intervening in the circadian time of infants may reduce their tendency to have negative temperaments.

In this study, frequent nighttime awakening in infants was not associated with their temperament after covariate adjustments. A previous cohort study by Hayes et al., which tracked infants from 6 weeks to 24 months of age, demonstrated statistical independence in the association between sleep-wake behavior and temperament [34]. This study showed a similar result even among one-month-old infants. The lack of association in our study might be because the frequency of infant awakening was self-reported by the mothers, which conforms to the statement by Hayes et al. in their study. Self-reporting by mothers may overlook quiet nighttime awakening in infants while the mothers are likely to be sleeping [34]. Conversely, some studies have shown that frequent awakenings or longer wake periods are associated with temperament [14,35]. Thus, no consistent results have been obtained, and further research is needed in the future.

In our study, sex differences in infants were observed in the association between longer daytime sleep than nighttime sleep periods and temperament. The associations with intense or frequent crying were significant among female infants in the logistic regression model. In particular, the rate of crying was significantly higher among female than male infants. The differences of sex in sleep duration and quality were reported in a previous study; at 6–115 months old, girls consistently slept longer (5–10 minutes) than boys. Male infants were also associated with shorter sleep periods among 14–27-month-old children [36–38]. A study suggested that difficulties in developing adaptive responses to environmental disturbances may be attributed to relatively slower development in male infants [38]. However, sex differences in temperament among one-month-old infants are still not apparent, and a biological cause likely underpins sex differences that affect sleep or temperament. Further studies are needed to elucidate the mechanism.

An association between infant sleep quality and temperament was observed among children cared for by women with or without prior childbirth experience. In particular, it was confirmed that prior childbirth experience in mothers was an effect modifier for the association between nighttime arousal and intense crying in children. This may be partly due to the differences between primiparae and multiparae delivery. Since different relationships were found depending on childbirth experience, it should be considered when considering interventions related to children’s sleep quality and temperament.

This study had some limitations. First, as JECS’s previous study mentioned, data on infant sleep and temperament were measured by maternal reports [20]. Parents often overestimated infants’ sleep time because of parental characteristics in data collection, such as education level, number of infants to care for, and level of sleep [39]. Therefore, certain infant sleep behavior may have been overlooked while the mother was unaware, and maternal characteristics can pose biases to the infant’s temperament. However, a study reported that parents’ report data agreed with polysomnography one [40]. Also, younger infants often require less intervention from parents; thus, our study parents’ reports may indicate lower levels of intervention than older infants’ parents’ reports [41]. Second, since this is a cross-sectional study, the temporal relationship between sleep and temperament in children cannot be clarified. Some inferences from previous studies can be made, but further intervention studies are needed to provide clear evidence of a relationship. Third, this was a large-scale study, and even minor differences may have been significant. Since there have been no similar studies, we believe that further research is needed. Fourth, this study may not have covered all aspects of infant temperament and sleep, such as failing to consider the “no crying” temperament. Despite these limitations, this study is the first large-scale study to investigate the association between sleep and temperament in infants in the first month of life, and is a valuable study with high generalizability.

## Conclusions

In conclusion, this study demonstrated that sleep problems were associated with temperament, such as frequent or intensive crying among one-month-old infants. Therefore, it is essential to focus on sleep disorders in early infancy to identify and prevent potential future developmental problems from a clinical and public health perspective. In addition, longitudinal studies are needed to elucidate how interventions to improve sleep disorders in infants may prevent future developmental problems.

## Supporting information

S1 Table Study participants data. (DOCX)
