# Supplementary material for: Association of sleep quality with temperament among one-month-old infants in The Japan Environment and Children’s Study
Source: PLoS One. 2022 Sep 14;17(9):e0274610. doi: 10.1371/journal.pone.0274610 (PMC9473436; doi:10.1371/journal.pone.0274610)
Supplement: S1 Table — (DOCX) [file pone.0274610.s001.docx]

**Study participants data**

|  |  | **Total population (n = 80,970)** | | **Five or more awakenings during the night** | | | | **Sleeping longer during the day than at night** | | | |
| --- | --- | --- | --- | --- | --- | --- | --- | --- | --- | --- | --- |
|  |  |  |  | **No (n = 75,812)** | | **Yes (n = 5,158)** | | **No (n = 65,354)** | | **Yes (n = 15,616)** | |
|  |  | **n^a^** | **(%)** | **n^a^** | **(%)** | **n^a^** | **(%)** | **n^a^** | **(%)** | **n^a^** | **(%)** |
| **Maternal characteristics** |  |  |  |  |  |  |  |  |  |  |  |
| Age at delivery (years) |  |  |  |  |  |  |  |  |  |  |  |
|  | < 25 | 354 | 6.9 | 7,472 | 9.9 | 354 | 6.9 | 6,073 | 9.3 | 1,753 | 11.2 |
|  | 25-29 | 1,357 | 26.3 | 21,150 | 27.9 | 1,357 | 26.3 | 18,215 | 27.9 | 4,292 | 27.5 |
|  | 30-34 | 1,943 | 37.7 | 26,854 | 35.4 | 1,943 | 37.7 | 23,400 | 35.8 | 5,397 | 34.6 |
|  | ≥ 35 | 1,504 | 29.2 | 20,336 | 26.8 | 1,504 | 29.2 | 17,666 | 27.0 | 4,174 | 26.7 |
| Parity |  |  |  |  |  |  |  |  |  |  |  |
|  | 0 | 1,839 | 35.8 | 33,381 | 44.2 | 1,839 | 35.8 | 27,112 | 41.6 | 8,108 | 52.1 |
|  | ≥ 1 | 3,293 | 64.2 | 42,146 | 55.8 | 3,293 | 64.2 | 37,991 | 58.4 | 7,448 | 47.9 |
| Smoking habits |  |  |  |  |  |  |  |  |  |  |  |
|  | Never smoked | 3,018 | 58.8 | 44,256 | 58.6 | 3,018 | 58.8 | 38,346 | 58.9 | 8,928 | 57.4 |
|  | Ex-smokers who quit before pregnancy | 1,294 | 25.2 | 17,652 | 23.4 | 1,294 | 25.2 | 15,323 | 23.5 | 3,623 | 23.3 |
|  | Smokers during early pregnancy | 824 | 16.0 | 13,634 | 18.1 | 824 | 16.0 | 11,447 | 17.6 | 3,011 | 19.4 |
| Marital status |  |  |  |  |  |  |  |  |  |  |  |
|  | Married | 4,919 | 96.4 | 71,653 | 95.6 | 4,919 | 96.4 | 61,930 | 95.8 | 14,642 | 94.8 |
|  | Unmarried | 156 | 3.1 | 2,719 | 3.6 | 156 | 3.1 | 2,195 | 3.4 | 680 | 4.4 |
|  | Divorced/widowed | 28 | 0.6 | 614 | 0.8 | 28 | 0.6 | 518 | 0.8 | 124 | 0.8 |
| Educational background (years) |  |  |  |  |  |  |  |  |  |  |  |
|  | < 10 | 210 | 4.1 | 3,388 | 4.5 | 210 | 4.1 | 2,824 | 4.4 | 774 | 5.0 |
|  | 10-12 | 1,470 | 28.8 | 23,369 | 31.2 | 1,470 | 28.8 | 19,924 | 30.9 | 4,915 | 31.8 |
|  | 13-16 | 3,349 | 65.6 | 46,997 | 62.8 | 3,349 | 65.6 | 40,791 | 63.2 | 9,555 | 61.9 |
|  | ≥ 17 | 78 | 1.5 | 1,129 | 1.5 | 78 | 1.5 | 1,012 | 1.6 | 195 | 1.3 |
| Household income (million Japanese-yen/year) |  |  |  |  |  |  |  |  |  |  |  |
|  | < 2 | 267 | 5.5 | 3,854 | 5.5 | 267 | 5.5 | 3,251 | 5.4 | 870 | 6.0 |
|  | 2 to < 4 | 1,549 | 32.1 | 24,132 | 34.4 | 1,549 | 32.1 | 20,595 | 34.0 | 5,086 | 35.3 |
|  | 4 to < 6 | 1,673 | 34.7 | 23,180 | 33.1 | 1,673 | 34.7 | 20,074 | 33.2 | 4,779 | 33.2 |
|  | 6 to < 8 | 756 | 15.7 | 11,322 | 16.1 | 756 | 15.7 | 9,915 | 16.4 | 2,163 | 15.0 |
|  | 8 to < 10 | 334 | 6.9 | 4,705 | 6.7 | 334 | 6.9 | 4,156 | 6.9 | 883 | 6.1 |
|  | ≥ 10 | 243 | 5.0 | 2,945 | 4.2 | 243 | 5.0 | 2,568 | 4.2 | 620 | 4.3 |
| Postpartum depressive symptoms at 1 month after delivery assessed by Edinburgh Postnatal Depression Scale | |  |  |  |  |  |  |  |  |  |  |
|  | No (score < 8) | 4,402 | 86.3 | 64,255 | 85.9 | 4,402 | 86.3 | 55,794 | 86.5 | 12,863 | 83.6 |
|  | Depressive (score ≥ 9) | 698 | 13.7 | 10,554 | 14.1 | 698 | 13.7 | 8,733 | 13.5 | 2,519 | 16.4 |
| Sleep duration during pregnancy (hours) |  |  |  |  |  |  |  |  |  |  |  |
|  | < 6 | 245 | 4.8 | 3,669 | 4.9 | 245 | 4.8 | 2,999 | 4.7 | 915 | 5.9 |
|  | 6 to <7 | 765 | 15.0 | 11,194 | 15.0 | 765 | 15.0 | 9,297 | 14.4 | 2,662 | 17.3 |
|  | 7 to <8 | 1,523 | 29.9 | 23,345 | 31.2 | 1,523 | 29.9 | 20,025 | 31.1 | 4,843 | 31.4 |
|  | 8 to <9 | 1,441 | 28.3 | 21,295 | 28.5 | 1,441 | 28.3 | 18,591 | 28.8 | 4,145 | 26.9 |
|  | 9 to <10 | 801 | 15.7 | 10,487 | 14.0 | 801 | 15.7 | 9,389 | 14.6 | 1,899 | 12.3 |
|  | >=10 | 324 | 6.4 | 4,842 | 6.5 | 324 | 6.4 | 4,202 | 6.5 | 964 | 6.3 |
| **Infant characteristics** |  |  |  |  |  |  |  |  |  |  |  |
| Gestational week |  |  |  |  |  |  |  |  |  |  |  |
|  | 37 | 575 | 11.2 | 7,213 | 9.5 | 575 | 11.2 | 6,006 | 9.2 | 1,782 | 11.4 |
|  | 38 | 1,385 | 26.9 | 17,266 | 22.8 | 1,385 | 26.9 | 14,748 | 22.6 | 3,903 | 25.0 |
|  | 39 | 1,564 | 30.3 | 22,372 | 29.5 | 1,564 | 30.3 | 19,462 | 29.8 | 4,474 | 28.7 |
|  | 40 | 1,264 | 24.5 | 21,466 | 28.3 | 1,264 | 24.5 | 18,623 | 28.5 | 4,107 | 26.3 |
|  | 41 | 370 | 7.2 | 7,495 | 9.9 | 370 | 7.2 | 6,515 | 10.0 | 1,350 | 8.6 |
| Small for gestational age |  |  |  |  |  |  |  |  |  |  |  |
|  | No | 4,751 | 92.6 | 70,062 | 92.8 | 4,751 | 92.6 | 60,465 | 92.9 | 14,348 | 92.2 |
|  | Yes | 381 | 7.4 | 5,465 | 7.2 | 381 | 7.4 | 4,638 | 7.1 | 1,208 | 7.8 |
| Infant sex |  |  |  |  |  |  |  |  |  |  |  |
|  | Male | 2,818 | 54.6 | 38,393 | 50.6 | 2,818 | 54.6 | 33,466 | 51.2 | 7,745 | 49.6 |
|  | Female | 2,340 | 45.4 | 37,419 | 49.4 | 2,340 | 45.4 | 31,888 | 48.8 | 7,871 | 50.4 |
| Feeding status |  |  |  |  |  |  |  |  |  |  |  |
|  | Breastfeeding | 3,366 | 66.4 | 39,134 | 52.8 | 3,366 | 66.4 | 34,893 | 54.6 | 7,607 | 49.9 |
|  | Partial breastfeeding | 1,627 | 32.1 | 32,103 | 43.3 | 1,627 | 32.1 | 26,699 | 41.8 | 7,031 | 46.1 |
|  | Formula feeding | 74 | 1.5 | 2,898 | 3.9 | 74 | 1.5 | 2,356 | 3.7 | 616 | 4.0 |
| Sleep duration (hours), median (IQR) |  | 15.5 (14-17) | | 15.5 (14-17) | | 15.5 (14.5-17) | | 15.5 (14-17) | | 15.5 (14-17) | |
| Sleep duration (hours) during the night (20:00 to 7:59), median (IQR) | | 8.5 (7.5-9.5) | | 8.5 (7.5-9.5) | | 8.5 (7.5-9) | | 8.5 (8-9.5) | | 7 (6-8) | |
| Sleep duration (hours) during the day (8:00 to 19:59), median (IQR) | | 7 (6-8) | | 7 (6-8) | | 7.5 (6.5-8.5) | | 7 (5.5-7.5) | | 8.5 (7.5-9) | |
| Number of awakenings during the night, median (IQR) | | 3 (2-3) | |  | n.a. |  | n.a. | 3 (2-3) | | 3 (2-3) | |
| ^a^Subgroup totals do not equal the overall number because of missing data. | | | | | | | | | | | |
| IQR:interquartile range; n.a: not applicable | | | | | | | | | | | |
